# Heterogeneity in RAG1 and RAG2 deficiency: 35 cases from a single-centre

**DOI:** 10.1093/cei/uxad110

**Published:** 2024-02-03

**Authors:** Betul Karaatmaca, Deniz Cagdas, Saliha Esenboga, Baran Erman, Cagman Tan, Tuba Turul Ozgur, Kaan Boztug, Mirjam van der Burg, Ozden Sanal, Ilhan Tezcan

**Affiliations:** Hacettepe University School of Medicine, Department of Pediatrics, Division of Pediatric Immunology, Ankara, Turkey; Department of Pediatric Allergy and Immunology, University of Health Sciences, Ankara Bilkent City Hospital, Ankara, Turkey; Hacettepe University School of Medicine, Department of Pediatrics, Division of Pediatric Immunology, Ankara, Turkey; Section of Pediatric Immunology, Institute of Child Health, Hacettepe University, Ankara, Turkey; Hacettepe University School of Medicine, Department of Pediatrics, Division of Pediatric Immunology, Ankara, Turkey; Section of Pediatric Immunology, Institute of Child Health, Hacettepe University, Ankara, Turkey; Section of Pediatric Immunology, Institute of Child Health, Hacettepe University, Ankara, Turkey; Hacettepe University School of Medicine, Department of Pediatrics, Division of Pediatric Immunology, Ankara, Turkey; St. Anna Children’s Cancer Research Institute (CCRI), Vienna, Austria; CeMM Research Center for Molecular Medicine of the Austrian Academy of Sciences, Vienna, Austria; Medical University of Vienna, Department of Pediatrics and Adolescent Medicine, Vienna, Austria; Ludwig Boltzmann Institute for Rare and Undiagnosed Diseases, Vienna, Austria; St. Anna Children’s Hospital, Vienna, Austria; Department of Pediatrics, Laboratory for Pediatric Immunology, Willem-Alexander Children's Hospital, Leiden University Medical Center, Leiden, The Netherlands; Hacettepe University School of Medicine, Department of Pediatrics, Division of Pediatric Immunology, Ankara, Turkey; Hacettepe University School of Medicine, Department of Pediatrics, Division of Pediatric Immunology, Ankara, Turkey; Section of Pediatric Immunology, Institute of Child Health, Hacettepe University, Ankara, Turkey

**Keywords:** autoimmunity, erythroderma, Omenn syndrome, RAG1/2, severe combined immunodeficiency, vasculitis

## Abstract

*Recombination activating genes* (RAG)1 and RAG2 deficiency leads to combined T/B-cell deficiency with varying clinical presentations. This study aimed to define the clinical/laboratory spectrum of RAG1 and RAG2 deficiency. We retrospectively reviewed the clinical/laboratory data of 35 patients, grouped them as severe combined immunodeficiency (SCID), Omenn syndrome (OS), and delayed-onset combined immunodeficiency (CID) and reported nine novel mutations. The male/female ratio was 23/12. Median age of clinical manifestations was 1 months (mo) (0.5–2), 2 mo (1.25–5), and 14 mo (3.63–27), age at diagnosis was 4 mo (3–6), 4.5 mo (2.5–9.75), and 27 mo (14.5–70) in SCID (*n* = 25; 71.4%), OS (*n* = 5; 14.3%), and CID (*n* = 5; 14.3%) patients, respectively. Common clinical manifestations were recurrent sinopulmonary infections 82.9%, oral moniliasis 62.9%, diarrhea 51.4%, and eczema/dermatitis 42.9%. Autoimmune features were present in 31.4% of the patients; 80% were in CID patients. Lymphopenia was present in 92% of SCID, 80% of OS, and 80% of CID patients. All SCID and CID patients had low T (CD3, CD4, and CD8), low B, and increased NK cell numbers. Twenty-eight patients underwent hematopoietic stem cell transplantation (HSCT), whereas seven patients died before HSCT. Median age at HSCT was 7 mo (4–13.5). Survival differed in groups; maximum in SCID patients who had an HLA-matched family donor, minimum in OS. Totally 19 (54.3%) patients survived. Early molecular genetic studies will give both individualized therapy options, and a survival advantage because of timely diagnosis and treatment. Further improvement in therapeutic outcomes will be possible if clinicians gain time for HSCT.

## Teaser

The recombination activating genes (RAG) are key players for T- and B-cell development and functions. Multifarious clinical presentations have been defined as patients with RAG deficiency. Early diagnosis, effective treatment, and early hematopoietic stem cell transplantation will increase the patients’ chances of survival, especially for late-onset forms.

## Graphical Abstract

**Graphical Abstract:**
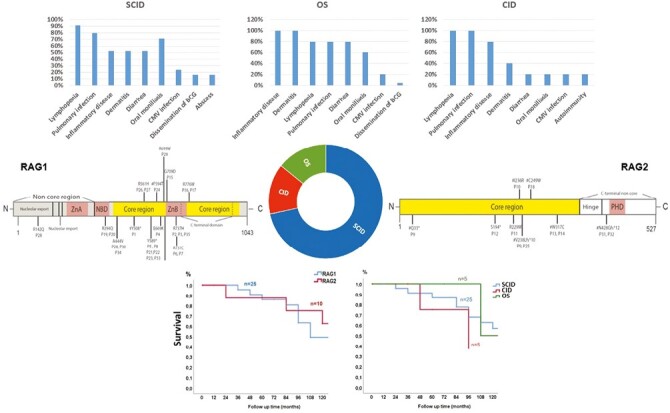

## Introduction

The recombination activating genes (RAG) 1 and 2 have essential roles in the early stage of V(D)J [variable (diversity) joining segments] recombination, which provides the plasticity of the adaptive immune system to give reaction to diverse antigens. Therefore, defect in the V(D)J recombination process leads to a restricted antigen receptor repertoire in the adaptive immune system [1].

Schwarz *et al*. [2] first described RAG gene mutations in patients with T-negative (T–), B-negative (B–), and natural killer cell-positive (NK+) severe combined immunodeficiency (SCID) in 1996. Further studies showed that human RAG gene mutations have a broad spectrum of clinical and immunological phenotypes other than classical SCID [3, 4]. In SCID patients, clinical findings usually begin in the first year of life, generally soon after birth. Life-threatening opportunistic viral and fungal infections are common. Patients experience recurrent sinopulmonary infections, interstitial pneumonitis, protracted diarrhea, and failure to thrive. Lymphopenia and severe hypogammaglobulinemia are frequent findings. Hematopoietic stem cell transplantation (HSCT) should be planned just after the diagnosis of SCID because T- and B-cell reconstitution is curative for SCID [5, 6].

A rare clinical presentation of RAG deficiency is Omenn syndrome (OS). RAG genes have a partial V(D)J recombination activity in OS [1, 7]. Omenn syndrome may result when certain hypomorphic RAG½ gene mutations result in partial V(D)J recombination activity, and leads to an activated oligoclonal T cell proliferation and infiltration in several organs, especially in skin, gut, and liver. The findings of OS are generalized erythroderma, lymphadenopathy, hepatosplenomegaly, eosinophilia, hypogammaglobulinemia, and high immunoglobulin (Ig) E levels. Clinical follow-up and treatment are similar to SCID [7, 8].

If hypomorphic RAG gene mutations are present, residual RAG protein activity is possible which will cause delayed-onset disease forms and mimic common variable immunodeficiency (CVID) or combined immunodeficiency (CID). In delayed-onset RAG deficiency patients, autoimmune cytopenia (AIC), vasculitis, nephritis, and granulomatous lesions in various tissues and organs are common in addition to recurrent sinopulmonary infections [9, 10]. Idiopathic CD4+ T-cell lymphopenia [11], IgA deficiency [12], selective deficiency of polysaccharide-specific antibody responses [13], and hyper-IgM syndrome [14] are other delayed-onset and atypical presentations. Different clinical phenotypes with the same RAG defect even in the same family may support the role of epigenetic factors on the phenotype [1].

Herein, we aimed to elucidate the clinical features, molecular diagnosis, and outcomes of RAG½ deficient patients followed by a tertiary pediatric immunology department over a twenty-year period by describing the variable clinical presentation.

## Material and methods

### Patients and study design

This study enrolled 35 RAGD patients from 30 families diagnosed in a twenty-year period (1999–2019) at Hacettepe University, Ihsan Dogramaci Children’s Hospital, Division of Pediatric Immunology. We retrospectively noted the clinical and laboratory data from medical records. We recruited the patients into three groups according to the clinical presentations/immunological findings; typical T(–)B(–)NK(+) SCID, OS, and delayed-onset CID (leaky SCID and atypical SCID) [9]. Hacettepe University Institutional Review Board approved the study, and the parents of the patients signed the informed consent.

SCID patients were diagnosed by using the European Society of Immunodeficiency Disorders (ESID) Criteria [15] and International Union of Immunological Societies (IUIS) guidelines [16]. OS criteria included the presence of erythroderma or atopic/seborrheic dermatitis in the absence of maternal engraftment [7, 17]. Delayed-onset patients with RAG½ mutations were diagnosed with delayed-onset CID, depending on the clinical symptoms and laboratory data [9].

The demographic characteristics of the patients (age of manifestation, age at diagnosis, gender, family history, etc.), clinical and laboratory findings, genetic mutations, HSCT outcomes, and survival were evaluated. RAG deficient patients those with high CD3 count, and high CD45RO value were assessed in terms of maternal engraftment, and karyotype and chimerism analyses were performed.

### Flow cytometry

We performed the analysis of peripheral blood lymphocyte populations by one laser three-color flow cytometry (BD Biosciences FACS Calibur, USA). One-hundred microliter of whole blood was obtained and stained with 20 μl of the monoclonal antibodies (CD3(fluorescein isothiocyanate (FITC)), CD4(FITC), CD8 (peridinin-chlorophyll protein complex (PerCP)), CD16 + 56(APC), and CD19 (phycoerythrin (PE)) (Beckton Dickinson, BD, USA)). Then, the samples were incubated in the dark for 15 min at room temperature.

### Sanger sequencing

DNA was isolated from peripheral blood mononuclear cells after separation using Ficoll-Paque (GE Healthcare, Little Chalfont, UK) according to the manufacturer’s instructions. Sequence analysis of RAG½ was performed following PCR amplification of the coding regions with TaqGoldTM (Life Technologies), followed by direct sequencing on an ABI Prism 3130 XL fluorescent sequencer (Applied Biosystems, Bleiswijk, the Netherlands).

### Targeted primary immunodeficiency panel screening

The molecular analyses of the patients were performed in the Hacettepe Pediatric Immunology Laboratory [18], Erasmus Center, and CeMM Research Center by using next-generation sequencing (NGS) for primary immune deficiency (PID) [19] and the Sanger Technique.

### Statistical analysis

Statistical analysis was performed by using SPSS^®^ version 22.0 for Windows (IBM SPSS, Chicago, IL, USA). Quantitative parameters were reported as means and SD, or as medians with 25th and 75th percentile values in case of skewed distribution. Categorical variables were described using absolute frequencies and proportions with a 95% CI. A *P*-value of <0.05 was considered statistically significant. Kaplan–Meier test was used for survival analysis.

## Results

### Patient characteristics

Thirty-five RAG-deficient patients (65.7% male) were included in the study. Eighty percent of cases had parental consanguinity, and 57.1% of the cases had a history of immunodeficiency in siblings or other family members. We subdivided the patients into three groups considering the clinical presentations and immunological findings: typical SCID (patients P1–25); OS (P26–30), and delayed-onset CID (P31–35) [9, 15, 16].

### RAG ½ mutations and affected domains

**Figure 1.**
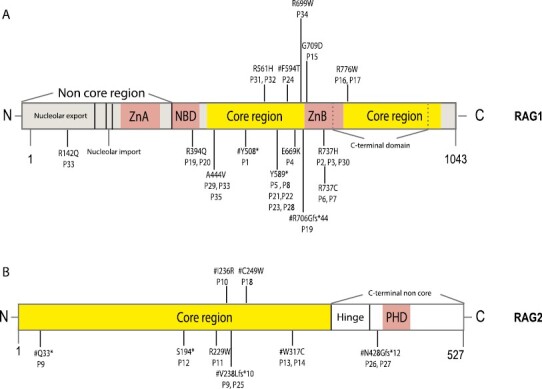
**A-B.** Mutations and affected RAG 1 and 2 domains in the patients. **#:** novel mutations. NBD: nanomer binding domain; PHD domain: the plant homeodomain; ZnA: zinc finger A; ZnB: zinc finger B

Twenty-five patients had RAG1, and 10 patients had RAG2 deficiency. The RAG½ mutations, and affected RAG½ domains are shown in Table 1 and Fig. 1A and B. Mutations were mostly found in the core region for RAG1 and RAG2 genes. **P26** and **P27** were cousins, and had novel RAG2 mutations affecting the C-terminal non-core domain.

**Table 1.** The analysis of the RAG mutations in our cohort

Patients	Gene	Variant	AA change	Variant type	Zygosity	Phenotype	Novelty (reference number)
**P1**	*RAG1*	c.1524T > C	Y508^*^	Nonsense	Hom	SCID	Yes
**P2, P3, and P30**	*RAG1*	c.2322G > A	R737H	Missense	Hom	SCID and OS	No [7]
**P4**	*RAG1*	c.2005G > A	E669K	Missense	Hom	SCID	No [20]
**P5, P8, P21, P22, P23, and P28**	*RAG1*	c.1879C > G	Y589^*^	Nonsense	Hom	SCID and OS	No [17]
**P6 and P7**	*RAG1*	c.2322C > T	R737C	Missense	Hom	SCID	No [21]
**P9**	*RAG2*	c.217C > T/c.712delG	Q33^*^/V238Lfs^*^10	Nonsense/Del. Frameshift	Comp. het.	SCID	Yes
**P10**	*RAG2*	c.707T > G	I236R	Missense	Hom	SCID	Yes
**P11**	*RAG2*	c.1886C > T	R229W	Nonsense	Hom	SCID	No [2]
**P12**	*RAG2*	c.1782C > A	S194^*^	Missense	Hom	SCID	No [21]
**P13 and P14**	*RAG2*	c.951G > T	W317C	Missense	Hom	SCID	Yes
**P15**	*RAG1*	c.2126G > A	G709D	Missense	Hom	SCID	No [22]
**P16 and P17**	*RAG1*	c.2326C > T	R776W	Missense	Hom	SCID	No [23]
**P18**	*RAG2*	c.746 G > A	C249W	Missense	Hom	SCID	Yes
**P19**	*RAG1*	c.1181G > A/c.2116delA	R394Q/R706Gfs^*^44	Missense/del. frameshift	Comp. het.	SCID	No [24]/Yes
**P20**	*RAG1*	c.1181G > A	R394Q	Missense	Hom	SCID	No [24]
**P24**	*RAG1*	c.1780_1781 delTTinsAC	F594T	Indel	Hom	SCID	Yes
**P25**	*RAG2*	c.712delG	V238Lfs^*^10	Del. frameshift	Hom	SCID	Yes
**P26 and P27**	*RAG2*	c.1280_1281insTGGATAT	N428Gfs^*^12	Ins. frameshift	Hom	OS	Yes
**P29** and **P35**	*RAG1*	c.1331C > T	A444V	Missense	Hom	OS and CID	No [17]
**P31** and **P32**	*RAG1*	c.1682G > A	R561H	Missense	Hom	CID	No [7]
**P33**	*RAG1*	c.537G > A/c.1443C > T	R142Q/A444V	Missense/Missense	Comp. het.	CID	No [25]/No [17]
**P34**	*RAG1*	c.2095C > T	R699W	Missense	Hom	CID	No [26]

AA: amino acid; *: stop codon, Hom: homozygous; Comp. het: compound heterozygous; Del: deletion; Ins: insertion.

All patients with RAG1 and RAG2 deficiency had homozygous mutations, except three patients (RAG 1 deficient **P19** and **P33** and RAG2 deficient **P9**) had compound heterozygous mutations. Among the thirty-five patients included in this study, three in the RAG1 gene and six in the RAG2 gene, a totally of nine novel mutations were reported and depicted in Table 1 and Fig. 1A and B. **P33** and **P34** were previously reported [25, 27].

### Clinical manifestations

**Figure 2.**
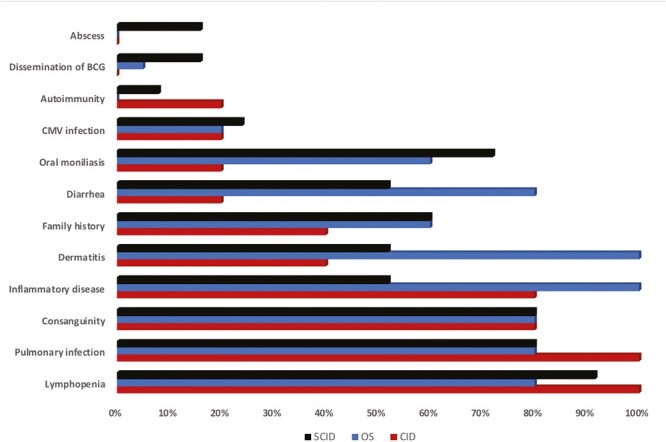
Common clinical manifestations of RAG deficient patients. Inflammatory disease: hepatomegaly and/or splenomegaly, lymphadenopathy

Common clinical manifestations were recurrent sinopulmonary infections 82.9%, oral moniliasis 62.9%, eczema/dermatitis 42.9%, diarrhea 51.4%, and autoimmunity 31.4% (Table 2 and Fig. 2).

**Table 2.** Characteristics of the RAG deficient patients

Patients	Presentation	Gender	Age of manifestation (months)	Age at clinical diagnosis (months)	Consanguinity	Family history	Clinical symptoms	Skin findings	Cytopenia	Other autoimmune/inflammatory disease	Vaccine- related disease
**P1**	SCID^**^	M	2	6	Yes	Yes	Diarrhea, pneumonia	No	No	HM and LAP	No
**P2** ^†^	SCID	M	0	3.5	Yes	Yes	Oral thrush, diarrhea, and RLRTI	No	No	HSM	No
**P3** ^†^	SCID	M	1	1	Yes	Yes	Pneumonia	Dermatitis	HA and ITP (after HSCT and DC–)	No	No
**P4**	SCID	M	4	7	No	No	RLRTI, RURTI, skin abscess, oral thrush, diarrhea, and USI	Dermatitis	No	HM	No
**P5**	SCID	M	4	6	Yes	Yes	Perianal abscess, diarrhea, and oral thrush	Dermatitis	No	HM and LAP	BCG lymphadenitis
**P6** ^†^	SCID	M	1	6	Yes	Yes	Oral thrush, diarrhea, pneumonia	No	No	No	No
**P7** ^†^	SCID	F	1.5	5	Yes	Yes	USI pneumonia	No	No	No	BCG lymphadenitis
**P8**	SCID	M	0.5	2	Yes	Yes	oral thrush, diarrhea	No	ITP (6 years after HSCT)	HM	No
**P9**	SCID	F	0	6	No	No	RLRTI, skin abscess, oral thrush, diarrhea, aphthous stomatitis	SD	No	No	BCG lymphadenitis
**P10**	SCID	F	1	25	Yes	No	RLRTI, skin abscess, oral thrush, and diarrhea	No	No	No	No
**P11**	SCID	F	1	10	Yes	No	CMV retinitis, oral thrush, diarrhea, and pneumonia	Dermatitis	ITP (CMV)	HM, LAP	No
**P12**	SCID	M	3	5	Yes	No	RLRTI and oral thrush	Whitish patches	No	HM	No
**P13**	SCID	F	1	3	No^*^	No	Oral thrush and pneumonia	No	AIHA (6 months after HSCT)	No	No
**P14**	SCID	M	1	7	Yes	No	RURTI, oral thrush, and aphthous stomatitis	DD	Leukopenia (bone marrow failure after HSCT)	HM	No
**P15**	SCID	M	2	3	Yes	No	Oral thrush and diarrhea	No	No	HM	No
**P16** ^*^	SCID	M	0	3	Yes	Yes	Oral thrush, RLRTI, and RURTI	No	No	No	No
**P17** ^*^	SCID	M	1	3	Yes	Yes	Oral thrush and CMV pneumonia	No	No	No	No
**P18**	SCID	M	6	11	Yes	Yes	RLRTI and RURTI	No	No	No	No
**P19**	SCID	M	2	2	No	Yes	RLRTI	Dermatitis	No	No	No
**P20**	SCID	M	1.5	4	Yes	No	RLRTI, oral thrush, diarrhea, and CMV retinitis	DD	No	No	No
**P21**	SCID	M	0.5	4	Yes	Yes	Oral thrush and pneumonia	No	Pancytopenia (bone marrow failure, after HSCT)	No	BCG lymphadenitis
**P22**	SCID	M	2	3	Yes	Yes	Diarrhea, pneumonia, oral thrush, and CMV pneumonia	DD	No	HM	No
**P23**	SCID	F	0.5	3	Yes	Yes	Diarrhea and oral thrush	DD	No	HM	No
**P24**	SCID	M	0.66	1.5	Yes	No	Pneumonia and USI	Dermatitis	No	No	No
**P25**	SCID	M	0	0.2	No	Yes		DD	No	No	No
**P26** ^†^	OS	F	0.5	4.5	Yes	Yes	Pneumonia and diarrhea	Ichthyosis and alopecia	No	HM	No
**P27** ^†^	OS	F	2	2.5	No	Yes	Pneumonia and diarrhea	Ichthyosis and alopecia	No	LAP	No
**P28**	OS	F	6	10	Yes	No	Oral thrush, RURTI, and pneumonia	SD, alopecia, and nail dystrophia	No	HM	No
**P29**	OS	F	4	9.5	Yes	No	Diarrhea, pneumonia, and oral thrush	SD	No	HM	No
**P30**	OS	F	2	2.5	Yes	Yes	RLRTI, oral thrush, diarrhea, and CMV pneumonia	SD, alopecia, and DD	No	HSM	No
**P31** ^*^	CID	M	7	78	Yes	Yes	RLRTI, bronhiectasis, and CMV pneumonia	Warts	No	LAP	No
**P32** ^*^	CID	F	36	62	Yes	Yes	RLRTI	Warts and vitiligo	No	LAP	No
**P33**	CID^**^	M	0.25	10	No	No	RLRTI, diarrhea, eczema, oral thrush, and CMV pneumonia	Granulomatous dermatitis	No	LAP	No
**P34**	CID	M	14	27	Yes	No	Pneumonia, hemoptysis, and pulmonary hemosiderosis	Necrotic wounds	No	Necrotizan vasculitis	No
**P35**	CID^**^	M	18	19	Yes	No	Candida cruzei pneumonia, RLRTI	No	No	Progressive neuropathy	No

**DC:** direct coombs; **DD:** diaper dermatitis; **HA:** hemolytic anemia **HM:** hepatomegaly; **HSM:** hepatosplenomegaly; **GIS:** gastrointestinal; **ITP:** immune thrombocytopenic purpura; **LAP:** lymphadenopathy; **RLRTI:** recurrent lower respiratory tract infections; **RURTI:** recurrent upper respiratory tract infections; **SD:** Seborrheic dermatitis; **USI:** urinary system infections.

^†^ **Cousins: P2** and **P3; P6** and **P7; P26** and P27;^*^: siblings: P16 and **P17**; **P31** and **P32**; **P1, P33,** and P35;^**^: Do not exactly fulfill the ESID criteria.

#### Autoimmune/inflammatory findings

Autoimmunity was recorded in 11 patients (31.4%); alopecia (*n* = 4), vitiligo (*n* = 2), granulomatous skin lesions and IBD (*n* = 1), vasculitis (*n* = 1), progressive neuropathy (*n* = 1), and AIC [AIHA (autoimmune hemolytic anemia), ITP (immune thrombocytopenia)] (*n* = 2, SCID patients post-HSCT). The ratio of AIC was 2/35 (6%) in RAG½ deficiency in this cohort.

Almost all autoimmune findings were generally associated with the CID group, albeit a patient with vitiligo was in the SCID group and patients with alopecia were in the OS group. (Table 2). Inflammatory disorders including hepatomegaly and/or splenomegaly, lymphadenopathy and several forms of dermatitis were quite common in all groups (Table 2/Fig. 2).

#### Infectious diseases

CMV infection developed in 8/35 patients (SCID = 6, CID = 1, and OS = 1), and in two SCID patients (**P11** and **P20**) retinitis developed as a complication. Immune thrombocytopenic purpura associated with CMV infection developed in **P11** at the age of 1.5 mo [2]. Foscarnet and ganciclovir were given. After referral, she was diagnosed with SCID and treated with HSCT successfully. The other SCID patient developed CMV retinitis during the disease course and underwent HSCT. Despite ganciclovir and CMV hyperimmunoglobulin, blindness developed.

Warts occurred in two siblings in the CID group (**P31** and **P32**); the lesions were resistant to cryotherapy and laser in one. They had a previously reported RAG1 mutation (R561H; c.1682 G > A) [7].

Bacillus-Calmette–Guérin (BCG) is a live-attenuated vaccine and is contraindicated in SCID patients. Unfortunately, it is administered soon after birth since tuberculosis is still a public health problem in some countries [28]. BCG is in the national vaccination schedule, and applied at the age of 2 mo in Turkey. As the median (IQR) age at diagnosis was 5 (3–10) mo in our cohort, 23 out of 35 patients received BCG vaccine before the diagnosis of PID. All BCG-vaccinated patients received isoniazid (INH) and rifampicin (RIF) for tuberculosis prophylaxis. Four SCID patients (**P5, P7, P9,** and **P21**) were diagnosed with BCGitis after HSCT and treated with additional anti-mycobacterial drugs.

### Laboratory findings

Lymphopenia (88.6%) was the most common laboratory finding (Table 3 [9, 29]), present in 92% of SCID patients (**P1**–**25**), 80% of OS (**P26**–**30**) patients, and 80% of CID (**P31**–**35)** patients. The definition of lymphopenia and the normal ranges of lymphocyte subsets used in this manuscript was based on the study of Shearer et al. [29].

**Table 3.** Laboratory findings of RAG deficient patients

Patients	WBC (/mm³)	ALC (/mm³)	ANC(/mm³)	AEC (/mm³)	IgA (mg/dl)	IgG (mg/dl)	IgM (mg/dl)	IgE (IU/l)	CD3^**^	CD4^**^	CD8^**^	CD16 + 56^**^	CD19^**^
**SCID**													
**P1**	8900	4806	1900	800	251 7–123	4090 304–1231	1 32–203	<1	17/49–76 817 1900–5900	11/31–56 528 1400–4300	21/12–24 1009 500–1700	79/3–15 3796 160–950	1/14–37 48 610–2600
**P2** ^†^	4100	984	2700	80	27 13.5–72	88 294–1165	70 33–154	N/A	1.8/51–77 18 2500–5600	1.9/35–56 19 1800–4000	41/12–23 403 590–1600	73.2/3–14 718 170–830	2.6/11–41 26 430–3000
**P3** ^†^	6300	1000	3300	1400	<6.67 11–14	402 633–1466	<4.17 22–87	<1	2/53–84 20 2500–5500	N/A	N/A	N/A	2/6–32 20 300–2000
**P4**	5400	1200	2800	500	9,7 7–123	11 304–1231	358.2 32–203	8.8	28/49–76 336 1900–5900	16.7 200 1400–4300	27/12–24 324 500–1700	57.7/3–15 692 160–950	0.2/14–37 2.4 610–2600
**P5**	3800	700	800	400	0 7–123	177 304–1231	2 32–203	<1	0/49–76 0 1900–5900	3/31–56 21 1400–4300	46/12–24 322 500–1700	79/3–15 553 160–950	0/14–37 0 610–2600
**P6** ^†^	4700	940	2500	0	<6.67 7–123	404 304–1231	<4.17 32–203	<1	0/49–76 0 1900–5900	7/31–56 66 1400–4300	32/12–24 300 500–1700	66.8/3–15 628 160–950	0/14–37 0 610–2600
**P7** ^†^	1100	300	400	0	18 13.5–72	120 294–1165	11 33–154	<1	0/51–77 0 2500–5600	3/35–56 9 1800–4000	44/12–23 132 590–1600	95/3–14 285 170–830	0/11–41 0 430–3000
**P8**	17000	1000	15600	300	8 13.5–72	580 294–1165	16 33–154		0/53–84 0 2500–5500	2/35–64 20 1600–4000	26/12–28 260 560–1700	57/4–18 570 170–1100	0/6–32 0 300–2000
**P9**	2800	400	1500	100	21 7–123	110 304–1231	22 32–203	<1	2/49–76 8 1900–5900	5/31–56 20 1400–4300	46/12–24 184 500–1700	86/3–15 344 160–950	0/14–37 0 610–2600
**P10**	3200	400	1700	100	21 26–296	510 604–1941	25 71–235	100	10/56–75 40 1400–3700	17/28–47 68 700–2200	33/16–30 132 490–1300	87/4–17 348 130–720	0/14–33 0 390–1400
**P11**	20300	10200	8932	100	47.3 17–107	641 463–1006	39 46–159	11.3	11.3/49–76 1153 1900–5900	7.38/31–56 752 1400–4300	24.5/12–24 2499 500–1700	70.8/3–15 7221 160–950	3.69/14–37 376 610–2600
**P12**	6300	1260	4400	500	35 13.5–72	120 294–1165	6 33–154	<1	0.9/51–77 11 2500–5600	1.8/35–56 24 1800–4000	41.5/12–23 529 590–1600	84.7/3–14 1071 170–830	0.7/11–41 10 430–3000
**P13**	4100	100	3000	0	24 13.5–72	420 294–1165	17 33–154	18.3	0/51–77 0 2500–5600	0.4/35–56 0.4 1800–4000	0.4/12–23 0.4 590–1600	93/3–14 93 170–830	0.4/11–41 0.4 430–3000
**P14**	100	0	0.1		<22 7–123	884 304–1231	19 33–154	<18	13/49–76 0 1900–5900	0/31–56 0 1400–4300	39/12–24 0 500–1700	57/3–15 0 160–950	0/14–37 0 610–2600
**P15**	2800	900	1400	0	<6.67 13.5–72	69.9 294–1165	<4.17 33–154	<1	0/51–77 0 2500–5600	1/35–56 9 1800–4000	16/12–23 144 590–1600	86/3–14 774 170–830	0/11–41 0 430–3000
**P16** ^*^	4600	800	2900	100	<6.67 13.5–72	136 294–1165	<4.17 33–154		0.1/51–77 0.8 2500–5600	51.1/35–56 1800–4000	25,2/12–23 201 590–1600	57,1/3–14 456 170–830	0.64/11–41 5 430–3000
**P17** ^*^	1700	200	1200	0	<6.67 13.5–72	755 294–1165	<4.17 33–154	<1	1/51–77 2 2500–5600	0/35–56 0 1800–4000	3/12–23 6 590–1600	9/3–14 18 170–830	4/11–41 8 430–3000
**P18**	11900	900	9300	700	225 17–69	1150 463–1006	168 46–159	935	16/49–76 144 1900–5900	13/31–56 117 1400–4300	27/12–24 243 500–1700	67/3–15 603 160–950	8/14–37 72 610–2600
**P19**	3400	400	2000	200	18 13.5–72	290 294–1165	8 33–154	<1	16/53–84 64 2500–5500	19/35–64 76 1600–4000	20/12–28 80 560–1700	64/4–18 256 170–1100	0.6/6–32 2.4 300–2000
**P20**	7700	1100	5400	0	<6.67 13.5–72	137 294–1165	<4.17 33–154	66	2/51–77 22 2500–5600	1/35–56 11 1800–4000	24/12–23 264 590–1600	65/3–14 715 170–830	2/11–41 22 430–300
**P21**	5100	900	2900	200	<6.67 13.5–72	102 294–1165	6.33 33–154	<1	0/51–77 0 2500–5600	73/35–56 63 1800–4000	13/12–23 117 590–1600	72/3–14 648 170–830	1/11–41 9 430–300
**P22**	7400	1100	4000	0	<6.67 13.5–72	191 294–1165	<4.17 33–154	<1	4/53–84 44 2500–5600	6/35–64 66 1800–4000	8/12–28 88 590–1600	66/4–18 726 170–830	4/6–32 44 430–300
**P23**	4600	800	3600	100	<6.67 13.5–72	262 294–1165	<4.17 33–154	<1	1/53–84 8 2500–5600	3/35–64 24 1800–4000	26/12–28 208 590–1600	85/4–18 680 170–830	0/6–32 0 430–300
**P24**	8400	1300	4500	1200	<6.67 9–30	367 376–685	44,1 36–77	43,1	36/53–84 468 2500–5500	36/35–64 468 1600–4000	26/12–28 338 560–1700	56/4–18 728 170–1100	0/6–32 0 300–2000
**P25**	11600	100	9600	500	<6.67 11–14	888 633–1466	<4.17 22–87	<1	64/53–84 64 2500–5500	10/35–64 10 1600–4000	54/12–28 54 560–1700	28/4–18 28 170–1100	0/6–32 0 300–2000
**OS**													
**P26** ^†^	25600	10240	3072	2560	17.7 13.5–72	126 294–1165	10.7 33–154	<1	9/49–76 921 2500–5600	9/31–56 921 1800–4000	23/12–24 2355 590–1600	83/3–15 8397 170–830	0/14–37 0 430–3000
**P27** ^†^	12200	1220	7320	1220	<6.67 13.5–72	226 294–1165	<4.17 33–154	<1	21/53–84 240 2500–5500	17/35–64 200 1600–4000	28/12–28 380 560–1700	68/4–18 850 170–1100	0/6–32 0 300–3000
**P28**	7200	2400	1400	500	11 17–69	61 463–1006	32 32–203	>1000	84.2/49–76 2016 1900–5900	41.1/31–56 984 1400–4300	52.7/12–24 1264 500–1700	13.5/3–15 324 160–950	0.5/14–37 12 610–2600
**P29**	20500	2240	15000	470	<30 17–69	<160 463–1006	<22 32–203	5	31/49–76 694 1900–5900	29/31–56 650 1400–4300	24/12–24 537 500–1700	53/3–15 1187 160–950	0/14–37 0 610–2600
**P30**	4900	500	1300	2600	<6.67 13.5–72	1180^***^ 294–1165	5.84 33–154	<1	16/51–77 80 2500–5500	17/35–64 200 1600–4000	24/12–23 120 560–1700	64/3–14 320 170–1100	2/11–41 10 300–3000
**CID**													
P31*	19700	1000	17500	100	208 70–303	1040 764–2134	150 69–387	5.37	45/60–76 450 1200–2600	19/31–47 190 650–1500	24/18–35 240 370–1100	46/4–17 460 100–480	8/13–27 80 270–860
P32*	5300	1000	3500	200	48,7 57–282	939 745–1804	154 78–261	14.4	21/56–75 210 1400–3700	13/28–47 130 700–2200	28/16–30 280 490–1300	40/4–17 400 130–720	8/14–33 80 390–1400
**P33**	4400	2600	1000	0	12 17–69	560 463–1006	11 46–59	<1	49/49–76 1274 1900–5900	36/31–56 936 1400–4300	9/12–24 234 500–1700	44/3–15 1144 160–950	1/14–37 26 610–2600
**P34**	10500	1000	9100	0	36.2 26–296	1020 604–1941	47.3 71–235	255	45/56–75 450 1400–3700	23/28–47 230 700–2200	35/16–30 350 490–1300	36/4–17 360 130–720	9/14–33 90 390–1400
**P35**	2200	300	900	0	11 30–107	14 605–1430	8 66–228	<1	40/53–75 120 2100–6200	21/32–51 63 1300–3400	32/14–30 96 620–2000	38/3–15 114 180–920	0/16–35 0 720–2600

^†^ **: cousins: P2** and **P3**; **P6** and **P7**; **P26** and **P27**; ^*^: siblings: P16 and **P17**; **P31** and **P32**; ^**^: **[%-/mm**^**3**^**)]**; ^***^: after IVIG treatment; N/A: not applicable.

**AEC**: Absolute eosinophil count; **ALC:** Absolute lymphocyte count; **ANC:** Absolute neutrophil count; **WBC:** White blood cell.

All of the labs were measured in the first visit.

Fifty-two percent of SCID patients, 80% of OS patients, and 20% of CID patients had low IgA, IgG, and IgM on admission. Normal/high IgG levels in some of the SCID patients were attributed to partially transplacental IgG transfer from their mothers. Most of the patients especially in the OS group had profound hypogammaglobulinemia on the first visit. Laboratory findings of RAG-deficient patients are summarized in Table 3.

### Classification of patients with RAG½ deficiency

#### Typical severe CID patients

Twenty-five patients, 19 males and 6 females were diagnosed with typical T(–) B(–) NK(+) SCID (patients [**P**] 1–25). The median age of clinical manifestations was 1 (0.5–2) mo and the age at diagnosis was 4 (3–6) mo. The parental consanguinity ratios were 15/17 and 5/8 in patients with RAG1 and RAG2 deficiency, respectively. Early onset of life-threatening infections and lymphopenia were common findings in SCID patients. Almost all patients except **P1** and **P11** [2] had lymphopenia [29]. Eczema and diaper dermatitis were also common. Clinical and laboratory characteristics are given in Tables 2 and 3.

#### OS patients

Five female patients were diagnosed with OS **(P26**–**P30). P26** and **P27** were cousins and had novel RAG2 mutations. The median age of clinical manifestations was 2 (1.25–5) mo, and the age at diagnosis was 4.5 (2.5–9.75) mo. All except one OS patient were born to consanguineous parents (**P27**’s parents were from the same village). Dermatitis was a common finding in all OS patients. Diffuse erythroderma, exfoliative dermatitis, and diffuse seborrheic dermatitis were present sometimes with alopecia and nail dystrophy. They had very low B-cell counts. Eosinophilia was present in 3/5. Only one patient **P28** had elevated IgE [17].

#### Delayed-onset CID patients

In this cohort, the ratio of hypomorphic defects was 5/35 (14.3%). All **(P31**–**P35**) were RAG1 deficiency patients with delayed-onset (CID). The median age of clinical manifestations was 14 (3.63–27) mo, and the median age at diagnosis was 27 (14.5–70) mo. The male/female ratio was 4/1. **P31** and **P32** were siblings presented with recurrent sinopulmonary infections and widespread warts [7]. **P33** had skin granuloma, and protracted diarrhea, mimicking inflammatory bowel disease (IBD) [25]. **P34** had isolated CD4 deficiency when he was admitted with hemoptysis and dyspnea due to pulmonary hemorrhage. He was diagnosed with polyarteritis nodosa (PAN) [26, 27]. Hemoptysis recurred, and Coombs (+) AIHA developed at 18 mo of age. Despite immunosuppressives (steroids, cyclophosphamide, and azathioprine) and supportive treatments, vasculitis deteriorated, digital necrosis, and autoamputation developed. **P35** was admitted with recurrent sinopulmonary infections and gingival hypertrophy at the age of 1.5 years [17]. Ataxia and progressive neurological deterioration developed when he was 25 mo old.

### Survival and outcome

Twenty-eight patients (80%) (SCID; 22, OS; 2, and CID; 4) underwent HSCT. Nineteen had an HLA-matched family donor, five had haploidentical (parent) donors, and four patients had a matched unrelated donor (MUD) (Table 4). Eleven out of 28 patients received pre-transplant conditioning before HSCT, those who did not receive were in the SCID group, and one in the OS group (Table 4). The median (IQR) age at HSCT was 7 (4–13.5) mo, and the success of HSCT was 67.9% (19/28). There was a significant difference in the median (IQR) age at HSCT among the clinical groups (*P* = 0.002). Median age at HSCT was 6 (3.5–9.9) mo in the SCID group, and it was 90.3 (51.4–115.3) months in the CID group. All except **P24** who did not receive pretransplant conditioning are alive and well after HSCT (16/17).

**Table 4.** Survival and outcomes of the RAG deficient patients

Patients	Presentation	HSCT/donor (HLA)	Age at HSCT (months)	Pre-transplant conditioning	GVHD	Complications	Outcome
**P1**	SCID^**^	Yes/sister (9/10)	7	No	Yes (grade 1/skin)	No	Alive
**P2** ^†^	SCID	Yes/father (10/10)	4	No	No	No	Alive
**P3** ^†^	SCID	Yes/mother (10/10)	1.5	No	Yes (grade 2/skin)	HA and ITP	Alive
**P4**	SCID	Yes/haplo (father) and haplo (mother)	16 and 25.5	Yes	No	Recurrent infections	Deceased
**P5**	SCID	Yes/haplo (father)	7	Yes	Yes (grade 3/skin and GIS)	PTLD	Alive
**P6** ^†^	SCID	Yes/haplo (mother)	9	Yes	No	Sepsis	Deceased
**P7** ^†^	SCID	Yes/sister (6/6)	5.5	No	Yes (grade 1–2/skin)	No	Alive
**P8**	SCID	Yes/sister (6/6)	2.5	No	Yes (grade 2/skin and liver)	ITP	Alive
**P9**	SCID	Yes/sister (10/10)	7 and 23	No	No	Booster HSCT (graft failure)	Alive
**P10**	SCID	Yes/sister (10/10)	26.5	No	No	No	Alive
**P11**	SCID	Yes/cousin (10/10)	11	No	No	CMV retinitis	Alive
**P12**	SCID	Yes/mother (10/10)	6	No	Yes (grade 1/skin)	Anemia and leukopenia	Alive
**P13**	SCID	Yes/haplo (father)	3.5	Yes	No	Treatment resistant AIHA	Deceased
**P14**	SCID	Yes/haplo (father)	13.5	Yes	No	Graft failure, sepsis, and neurologic complications	Deceased
**P15**	SCID	Yes/cousin (10/10)	6	No	No	No	Alive
**P16** ^*^	SCID	Yes/MUD (9/10)	9.5	Yes	No	Pneumonia	Deceased
**P17** ^*^	SCID	No		N/A			Deceased
**P18**	SCID	Yes/sister (10/10)	12.5	No	No	No	Alive
**P19**	SCID	No		N/A			Deceased
**P20**	SCID	Yes/sister (10/10)	4.5	No	Yes (grade 1/skin)	Blinded by CMV retinitis	Alive
**P21**	SCID	Yes/mother (10/10)	5	No	Yes (grade 2/skin and liver)	Pancytopenia and immunosuppressive therapy due to chronic GVHD	Alive
**P22**	SCID	Yes/sister (10/10)	3.5	No	Yes (grade 1/skin)	Acute GVHD	Alive
**P23**	SCID	No		N/A			Deceased
**P24**	SCID	Yes/mother (10/10)	3	No	Yes (isolated liver)	Isolated liver GVHD and pancytopenia	Deceased
**P25**	SCID	Yes/brother (10/10)	3.5	No	Yes (grade 1/skin)	No	Alive
**P26** ^†^	OS	No		N/A			Deceased
**P27** ^†^	OS	No		N/A			Deceased
**P28**	OS	Yes/mother (5/6)	24	Yes	Yes (grade 1-2/skin/GIS and liver)	Abducens palsy and pneumonia	Deceased
**P29**	OS	Yes brother (10/10)	10	No	No	No	Alive
**P30**	OS	No		N/A			Deceased
**P31** ^*^	CID	Yes/sister (10/10)	121	Yes	No	No	Alive
**P32** ^*^	CID	Yes/MUD (10/10)	98	Yes	No	No	Alive
**P33**	CID^**^	No		N/A			Deceased
**P34**	CID	Yes/MUD (9/10)	82.5	Yes	No	Pneumonia and acute kidney failure	Deceased
**P35**	CID^**^	Yes/MUD (cord blood,10/10)	41	Yes	No	Bronchiolitis obliterans organizing pneumonia	Deceased

**HA:** hemolytic anemia; **HLA:** human leukocyte antigen; **ITP:** immune thrombocytopenic purpura; **MUD:** Match unrelated donor; **N/A:** Not applicable; **PTLD:** Post-transplant lymphoproliferative disorders.

^†^ **: cousins: P2** and **P3; P6** and **P7**; **P26** and **P27**.

^*^ **: siblings**: **P16** and **P17**; **P31** and **P32**.

**P1, P33** and P35^**^: Do not exactly fulfill the ESID criteria.

Twelve patients (SCID; 11, OS; 1) received immunosuppressive treatments (methylprednisolone and cyclosporine) for GVHD (Table 4). **P21** with a previously reported homozygous RAG1 mutation (Y589*; c.1879C > G) [17] underwent HLA-identical HSCT from his mother at 4 mo of age. A liver biopsy for persistent transaminase elevation revealed GVHD. MPZ and cyclosporine (CYC) were given. Skin exfoliation, thickening, excoriation, and pancytopenia suggest bone marrow failure developed despite the treatment. Afterwards, he was diagnosed with chronic GVHD. He is under tacrolimus and mycophenolate mofetil (MMF) treatments for chronic GVHD. **P24** had a novel RAG1 mutation and was diagnosed with isolated liver GVHD and treated with CYC, MMF, and etoposide. Liver functions deteriorated and progressive liver failure developed despite plasmapheresis and mesenchymal stem cell transplantation. All other patients with acute GVHD were treated with MPZ and/or CYC (Table 4).

Autoimmune cytopenia developed in two SCID patients after HSCT. **P8** developed idiopathic thrombocytopenic purpura (ITP) 6 years after HSCT [17], and he was successfully treated with intravenous immunoglobulin (IVIG) therapy. Autoimmune hemolytic anemia developed 6 mo after HSCT in **P13** with a novel RAG2 mutation. Unfortunately, despite the treatment [IVIG, pulse steroids, plasmapheresis (three times), CYC, cyclophosphamide, rituximab, and MMF] and supportive care for persistent AIHA, the patient died before the second HSCT planned from another HLA-matched sibling donor.

**Figure 3:**
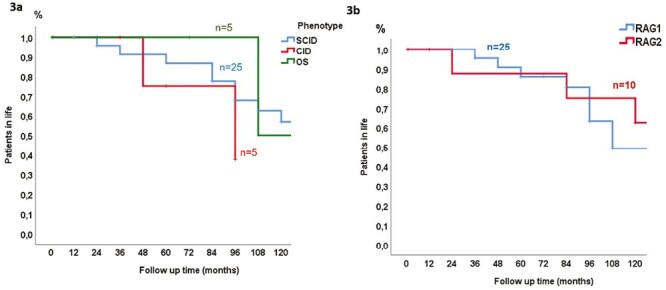
**A.** The survival analysis of the three distinct clinical groups. **B.** The survival analysis according to the type of RAG deficiency

In total, 16 patients died during the disease course, including nine patients who underwent HSCT (SCID; 6, OS; 1, and CID; 2). Nineteen patients (54.3%) are alive, and well after HSCT. The 10-year-survival analysis is shown in Fig. 3A for distinctive clinical groups, and in Fig. 3B according to the type of RAG deficiency. Survival differed in the groups; it was maximum in the SCID patients (64%) who mostly had an HLA-matched family donor, and minimum in the OS patients (20%) only P29 survived after a successful HSCT with the full-matched family donor. (Table 4). There was no difference between RAG1 and RAG2 deficient SCID patients in terms of HSCT outcomes, autoimmunity, and survival (*P* > 0.05).

## Discussion

Here, we present a large cohort of RAG ½ deficient patients (25 patients with RAG1, 10 with RAG2 deficiency) during a 20-year period from Turkish origin with nine novel mutations. In our study patients were classified as SCID, OS, and CID, and it was also depicted that identical mutations can cause distinct clinical presentations. All novel mutations except one caused SCID phenotype. We believe that the identified novel variants in this study can contribute to the literature, and help to understand the nature of the RAG deficiency.

RAG deficiency was described in various studies from all over the world with different clinical pictures from the first cases until today with raising awareness. RAG mutations were particularly reported from highly consanguineous populations for instance Middle East region [30–32]and Turkey [33, 34], whereas there were also large case series from the Slavic countries [35], Italy [36] and Latin America [37] in which consanguineous marriages seen relatively less common. Noteworthy, similar mutations have been reported from different ethnic origins requiring more research.

Due to the high rate of consanguinity in our population [38, 39], we had a higher rate of homozygous mutations compared to other European nations [36]. RAG½ deficiency is the predominant genetic reason for SCID phenotype in Turkey, and the reported frequency among studies varies between 15.4% and 26% [6, 40]. Furthermore, in the present study, most of the patients had SCID phenotype similar to the studies from the Middle East region [30, 32], and in contrast to Slavic [35] and Italian [36] cohorts in which OS was more prevalent.

The RAG½ gene mutations have a broad spectrum of phenotypes, ranging from SCID, OS, and delayed-onset CID/AS. The RAG deficient patients with SCID and OS generally present with opportunistic infections in early infancy. Diagnosis of delayed-onset CID due to hypomorphic RAG½ deficiency is more challenging due to clinical variation. In some patients, the diagnosis may not be possible [9, 41].

A single mutation may result in a variety of clinical manifestations [7, 13, 16]. Patients with the same RAG mutation may have different phenotypes even in the same family [42], possibly due to epigenetic factors including gene modifiers, environmental factors, infections, and iatrogenic factors [43]. Furthermore, researchers showed that similar mutations in the N-terminal truncation of the RAG1 protein cause different RAG residual protein activity, which leads to distinct clinical phenotypes [44].

The published studies regarding RAG1 and RAG2 deficiencies indicated that more than 60 RAG1 and RAG2 mutations are located in the core regions of the RAG proteins and they affect DNA binding, catalytic activity, or protein stabilization [45]. The core region mutations in our study also comprised the majority of the identified variants. In addition, two patients in our study with OS had a non-core region variant like in the articles of Grazzini *et al*. [46] and Matthews *et al*. [47]. These OS patients had severe ichthyosis-like skin lesions and alopecia, and unfortunately deceased before HSCT.

We observed an overall distribution of the causative variants including different types of monoallelic or biallelic variations located in different regions of the RAG1 and RAG2 genes. In addition, we did not detect a founder variant like in the study reported from Slavic countries [35]. Although the consanguinity rate is high among our patients, we think that they are coming from different regions of the country.

In our study group, most of the patients had RAG1 mutations in line with the literature [35, 36]. Interestingly, the majority of the novel mutations were RAG2 mutations presenting with SCID phenotype. P13 and P14 in the SCID group had the same novel homozygous missense mutation in the RAG2 protein core region, which is proposed to disturb the interaction with RAG1 and recombination signal sequence (RSS) and leads to RAG2 c.2152G > T mutation causing p.Trp317Cys. The tryptophan at this position is essential for interaction with RAG1 and cleavage of the DNA and the RSS [48, 49].

In the present study, recruiting some of the patients (**P1, P33,** and **P35**) to a clinical group according to the ESID criteria was challenging. Other parameters and clinical characteristics were indicative in grouping. The estimated prevalence of RAG½ mutations, leading to partial enzyme activity and a later presentation varies between 1% and 1.9% in adult PID cohorts [50]. An important finding of this cohort is that the ratio of hypomorphic defects was shown to be 5/25 (20%) for RAG1 deficiency.

Granulomatous diseases were first identified in three patients with compound heterozygous RAGD mutations [10]. Granulomatous lymphocytic interstitial lung disease (GLILD) may be associated with RAGD [51]. Granulomatous skin lesions were present in **P33**, a delayed-onset CID patient, who had previously reported compound heterozygous RAG1 mutations (c.537G > A/ c.1443C > T; R142Q/A444V) [17, 25].

Treatment-resistant severe vasculitis was present in **P34** and complicated with digital necrosis [26, 27]. He had a relatively delayed-onset CID caused by a homozygous RAG1 mutation (c.2095C > T; R699W). Similarly, in our study vasculitis was reported in RAG deficient patients. Henderson et al. described an early-onset autoimmune disease, Coombs (+) AIHA and vasculitis, causing digital necrosis, in a compound heterozygous RAG1 deficiency (c.2522 G > A; c.2920 T < C) [52]. Another compound heterozygous RAG1 deficiency patient again with a compound heterozygous defect (c.125A > G, M1V; c.2322 G > A, R737H) again presented with recurrent cutaneous vasculitis **[13]**. Partial RAG deficiency with vasculitis was reported in another study in six patients [53].

More than half of our patients (SCID; *n* = 13, OS; *n* = 4 and CID; *n* = 1) had a history of intractable diarrhea, a common symptom in SCID patients. It may present with IBD-like disease, autoimmune enteropathy, duodenitis, or severe noninfectious diarrhea. Detected infective agents are *pneumocystis jirovecii, Candida* species, and viral infections, such as cytomegalovirus (CMV) and adenovirus [54].

Viral infections are an important cause of morbidity and mortality in the course of RAG deficiency and are challenging for patients. Varicella infections, complicating with subsequent pneumonitis and ITP were reported in RAG-deficient patients [55, 56]. Another accompanying viral infection is CMV, which may progress to retinitis in PID patients. Early suspicion and effective treatment are crucial to prevent visual morbidity and loss in CMV retinitis [57, 58]. Two siblings (**P31** and **P32**) diagnosed with CID presented with widespread warts in our cohort. Efficient cellular and cytotoxic immunity provided by T and NK cells is necessary to cope with HPV infections [59].

A wide range of autoantibodies, anti-cytokine antibodies, and neutralizing antibodies against interferon-α and interferon-ω, may develop in RAG-deficient patients following viral infections [56, 60]. A meta-analysis showed that autoimmunity and inflammatory diseases developed in 67.1% of 134 RAG deficiency. Autoimmune and inflammatory diseases have been reported in delayed-onset CID patients, whereas they were rare in OS and SCID patients [41]. Autoimmune cytopenia, granuloma, skin cancer, vasculitis, neuropathy, interstitial lung disease, and myopathy were detected in 76.2% of patients with RAG1, and 23.8% of the patients with RAG2 deficiency [41].

In our study, AIC developed after the HSCT was performed without a conditioning regimen **(P8** and **P13**). Autoimmune cytopenia following HSCT, especially AIHA, was considered a serious post-HSCT complication with a poor prognosis [61]. Viral infections usually precede the onset of AIC [41]. The ratio of AIC was 2/35 (6%) in RAG½ deficiency in this cohort.

In the present study, 80% of all RAG½ patients who underwent HSCT had a survival rate of 54.3%. The median age at HSCT was 7 (4–13.5) mo, and the HSCT success in the RAG½ deficiency SCID group was 72.7% (16/22), a higher outcome than the general SCID–HSCT outcome (65.7% survival rate over 20 years) in Turkey [6]. Severe pneumonia was the leading cause of death in patients after HSCT. All RAG-deficient patients were diagnosed with SCID in a recently published study from Israel and the HSCT success rate was 68% [32]. The lack of newborn screening has a negative impact on the survival of our study patients, because, it causes a delay in both the PID diagnosis and timely HSCT.

In conclusion, we evaluated a considerable number of RAGD patients and identified certain novel mutations. A high proportion of patients presented with classical SCID phenotype. Early diagnosis, which will be accomplished after national neonatal screening, could improve clinical outcomes and survival. Patients with the lowest survival ratio, the delayed onset/CID patients, were the patients with the most frequent ratio of autoimmune/inflammatory findings. Thus, patients with autoimmunity and inflammation, including vasculitis, should be referred to immunology clinics and evaluated for delayed onset/CID. Early molecular diagnosis may also help in timely management. Definite and individualized therapeutic interventions which could only be possible after early diagnosis will provide a survival advantage, especially for delayed onset/CID patients until HSCT.

## Data Availability

All data are incorporated into the article and its online supplementary material.

## Abbreviations

AIC: autoimmune cytopenia
AIHA: autoimmune hemolytic anemia
BCG: Bacillus-Calmette–Guérin
CD: cluster of differentiation
CID: combined immunodeficiency
CID-G/AI: combined immunodeficiency with granulomas and/or autoimmunity
CMV: cytomegalovirus
CVID: common variable disease
GVHD: graft versus host disease
HLA: human leukocyte antigen
HSCT: hematopoietic stem cell transplantation
IBD: inflammatory bowel disease
Ig: immunoglobulin
IQR: interquartile ranges
IVIG: intravenous immunoglobulin
ITP: immune thrombocytopenia
MMF: mycophenolate mofetil
NGS: next generation sequencing
NK: natural killer
OS: Omenn syndrome
PID: primary immune deficiency
RAG½: recombination activating gene ½
RSS: recombination signal sequence
SCID: severe combined immunodeficiency.
